# Application of Kern’s framework to development of a family integrated care curriculum for neonatal intensive care units

**DOI:** 10.5116/ijme.6566.4b2c

**Published:** 2023-12-27

**Authors:** Pilar Zanoni, Karen M. Benzies, Deborah A. McNeil

**Affiliations:** 1 Faculty of Nursing, University of Calgary, Canada; 2 Alberta Health Services, Maternal Newborn Child and Youth Health, Strategic Clinical Network, Canada

**Keywords:** Kern’s framework, family integrated care curriculum, neonatal intensive care units

## To the Editor

Globally, the standard of care in NICUs is rapidly changing to a family integrated care (FICare) approach. FICare is an enhancement to family centred care (FCC) that integrates parents in the neonatal care team with education and support from healthcare providers.^1^^-^^5^

There is overwhelming evidence for the positive effects of FICare,^5^^-^^12^ which has been linked to infant outcomes of: (1) reduced length of stay without concomittant increases to emergency department visits or readmissions,^6^ (2) increased weight gain velocity,^5^^,^^7^ (3) increased breastfeeding rates,^5^^,^^7^ (4) decreased duration of supplemental oxygen,^7^ (5) decreased nosocomial infection rates, (6) decreased antibiotic exposure, (7) earlier skin-to-skin, (8) quicker achievement of full enteral feeds, and (9) less time on parenteral nutrition. Parent outcomes include decreased psychosocial distress^5^^-^^7^ and increased confidence.^6^ For the health system, FICare avoids cost.^7^^,^^12^ Despite these benefits, variability in its operationalization remains, which may be attributed to ambiguity of what constitutes FICare. The term has been used in reference to several initiatives involving parent empowerment and integration into the care team.^3^^,^^13^^-^^17^ Furthermore, FICare has been described as a model,^3^^,^^5^^,^^6^^, ^^14^^,^^18^^,^^19^ program,^20^^,^^21^ ethos,^22^ and philosophy of care.^23^^,^^24^ An ethos or philosophy of care represent values and guiding principles and may be broadly interpreted, as has been reported with Patient and Family Centered Care (PFCC).^25^^-^^32^ Conversely, a model or program are practical approaches that provide guidance and direction on how to successfully deliver services and measure its performance.^33^^,^^34^ Operationalization of FICare requires coherence between its characteristics and the practices of providers. Earlier work by our group reported that healthcare providers have a critical need for conceptual and experiential education about FICare.^37^ While staff education appears to be a necessary condition supporting implementation of FICare, to date, only one nursing education program to support FICare has been described.^36^

We used Kern’s Framework to develop the Alberta FICare curriculum for multidisciplinary neonatal care providers. Alberta FICare is a model of care that integrates parents into the care of their infant from the time of admission to the NICU. With practical tools and strategies, multidisciplinary neonatal care providers’ roles broaden with a focus on educating and supporting parents as they gain knowledge, skills, and confidence in the care of their infant.^6^ Kern’s 6-step approach to curriculum development has been applied successfully across multiple specialties^38^^-^^42^ and includes (1) problem identification and general needs assessment, (2) targeted needs assessment, (3) goals and objectives, (4) educational strategies, (5) implementation, and (6) evaluation and feedback.

Our general needs assessment (step 1) identified that although PFCC is espoused by healthcare systems, as a philosophy, guidelines for PFCC are limited by weak evidence^43^ and concepts remain fundamentally misunderstood by healthcare providers and families.^44^^-^^46^ A review of the literature showed that educational studies describing training for PFCC practices and FICare in NICUs were lacking.

The targeted needs assessment (step 2) began prior to initiating a cluster randomized controlled trial of Alberta FICare in Alberta, Canada. Interviews with healthcare providers and hospital administrators in level II NICUs found that the complexity of the health system interfered with good intentions and capacity to provide PFCC.^47^ These findings echoed a 2014 report from the Canadian Premature Infants Foundation.^48^ Policies play a significant role in parental access to NICU and participation in neonatal care. Within Alberta’s (Canada) health system, Alberta Health Services (AHS), provincial neonatology policies and guidelines are focused on administration, safety, and clinical care in the areas of transfer, skin assessment, and developmental care, among other things. While a provincial family presence policy^49^ sets the standard for 24/7 family presence in all care settings, there is variation in NICU-specific family presence policies. Rooming in policies and care-by-parent guidelines^49^ are typically limited to supporting parents whose infant will be discharged soon. Indeed, in a post implementation evaluation of Alberta FICare,^37^ healthcare providers spoke about the need for provincial supports to facilitate parental presence and integration in the NICU.

The goal (step 3) of Alberta FICare training is to teach multidisciplinary neonatal care providers to integrate families into the NICU care team starting from time of admission. To support the FICare change in culture, neonatal care providers receive training in three areas: (1) Relational Communication,^13^ (2) Parent Education, and (3) Parent Support. Curriculum objectives address six CANMeds roles (Communicator, Professional, Scholar, Collaborator, Health Advocate, Leader) and were designed to be specific and measurable across cognitive, affective, and psychomotor domains.

The educational content and strategies (step 4) were selected based on the training objectives and developed using adult learning theory.^50^^-^^52^ Our curriculum is composed of formal, eLearning modules and peer-reviewed journal articles describing the evidence that underpins each component of Alberta FICare. Alberta FICare training translates theoretical concepts to practical strategies and tools. eLearning modules include learning objectives, reflections on experience, activities with immediate feedback, clinical scenarios as examples of Alberta FICare in practice, links to peer-reviewed supporting evidence, and quizzes (passing mark requirement of 80%). Given shift work and busy clinical schedules, training is online and asynchronous to increase cognitive efficiency and minimize demands on learner’s attention.^53^ Providers complete one of two levels of training: (1) Super-Users, who are designated frontline leaders, and (2) End-Users, which includes all multidisciplinary neonatal care providers. It is ultimately the changes in provider practices that (1) enable integration of parents into their infant’s NICU care team, (2) standardizes delivery of evidence-informed education, and (3) facilitates psychosocial support for parents. eLearning modules with scientific and andragogically rigorous content are key to provider engagement, reflection, and anticipated adoption into practice.

Implementation (step 5) of eLearning was embedded in the provincial scale and spread of Alberta FICare in 14 NICUs in Alberta,^54^ co-sponsored by Alberta Health and AHS. Alberta FICare demonstrated a positive return on investment for the health system by freeing up costly NICU capacity through reduced length of stay without concomitant increases to emergency department visits or readmissions.^12^ Target learners for our curriculum are multidisciplinary neonatal care providers (physicians [neonatologists, pediatricians, clinical assistants], nurses, and allied health) and their trainees in NICU. The curriculum content was piloted, refined, and eLearning modules were embedded in the learning management systems (LMS) of AHS and its contractor, Covenant Health. NICUs in Alberta have included Alberta FICare training as part of orientation for new nursing hires, as well as fellows and residents. Curriculum content is reviewed annually and updated with emerging evidence.

Curriculum evaluation and learner feedback (step 6) is captured via a secure web-based tool to inform annual improvements to the training.

To the best of our knowledge, this curriculum is the only FICare training that (1) was developed using Kern’s framework, (2) targets multidisciplinary NICU staff (physicians, nursing, and allied health), and (3) is accredited for continuing medical education.

Currently, Alberta FICare training is accessible in the province of Alberta. Given demand from neonatal intensive care managers across Canada, efforts are underway to make Alberta FICare training and supporting implementation processes available to neonatal care teams nationally and internationally. Furthermore, adaptation and evaluation for inpatient pediatric settings is underway.

### Acknowledgements

The Alberta FICare Project Team for scale and spread wishes to acknowledge Senior Operating Officers, Executive Directors, Medical Directors, Patient Care Managers, Unit Managers, frontline healthcare providers, and parents for their support of the Alberta FICare scale and spread quality improvement initiative.

### Conflict of Interest

PZ received salary from the Health Innovation and Implementation (HIIS) Fund for the submitted work and is an employee of Liminality Innovations Inc, a social enterprise working to ensure that Alberta FICare (also known as Merge™) is accessible to NICUs across Canada and internationally. KMB is the CEO and founder of Liminality Innovations Inc. DAM declares no competing interests.
